# Intercellular signaling between ameloblastoma and osteoblasts

**DOI:** 10.1016/j.bbrep.2022.101233

**Published:** 2022-02-18

**Authors:** Elissa Chairani, Takao Fuchigami, Hirofumi Koyama, Yusuke Ono, Mikio Iijima, Michiko Kishida, Toshiro Kibe, Norifumi Nakamura, Shosei Kishida

**Affiliations:** a Department of Biochemistry and Genetics, Kagoshima University Graduate School of Medical and Dental Sciences, 8-35-1 Sakuragaoka, Kagoshima, 890-8544, Japan; b Department of Oral and Maxillofacial Surgery, Kagoshima University Graduate School of Medical and Dental Sciences, 8-35-1 Sakuragaoka, Kagoshima, 890-8544, Japan; c Department of Dentistry, Faculty of Medicine, Diponegoro University, Jl. Prof. Soedarto, Tembalang, Semarang, Central Java, 50275, Indonesia

**Keywords:** Ameloblastoma, AM-3, Osteoblast, Tumor-bone microenvironment, Cytokine, Intercellular communication

## Abstract

Ameloblastoma is an odontogenic tumor located in the bone jaw with clinical characteristics of extensive bone resorption. It is a locally invasive tumor with a high recurrence rate despite adequate surgical removal. In bone disease, tumors and other cells including osteoblasts, osteoclasts, and osteocytes in the bone microenvironment contribute to the pathogenesis of tumor growth. However, the effect of osteoblasts on ameloblastoma cells is not well-understood, and there has been limited research on interactions between them.

This study investigated interactions between ameloblastoma cells and osteoblasts using a human ameloblastoma cell line (AM-3 ameloblastoma cells) and a murine pre-osteoblast cell line (MC3T3-E1 cells). We treated each cell type with the conditioned medium by the other cell type. We analyzed the effect on cytokine production by MC3T3-E1 cells and the production of MMPs by AM-3 cells. Treatment with AM-3-conditioned medium induced inflammatory cytokine production of IL-6, MCP-1, and RANTES from MC3T3-E1 cells. The use of an IL-1 receptor antagonist suppressed the production of these inflammatory cytokines by MC3T3-E1 cells stimulated with AM-3-conditioned medium. The MC3T3-E1-conditioned medium triggered the expression of MMP-2 from AM-3 cells. Furthermore, we have shown that the proliferation and migration activity of AM-3 cells were accelerated by MC3T3-E1 conditioned media.

In conclusion, these intercellular signalings between ameloblastoma cells and osteoblasts may play multiple roles in the pathogenesis of ameloblastoma.

## Highlights

• Ameloblastoma conditioned medium induced IL-6, MCP-1 and RANTES production from osteoblast cell in IL-1 dependent manner.
• Unidentified factors from osteoblast conditioned medium induced MMP-2 production and stimulate proliferation and cellular motility of ameloblastoma cells.

## 1 Introduction

Recent studies showed that many properties and cell types within the bone microenvironment contribute to the pathogenesis of tumor growth in bone disease [1,2]. Osteoblasts acted as a potent chemoattractant for metastatic tumor cells of breast and prostate cancer. Metastatic cancer cells suppress the proliferation and adhesion of osteoblasts, which may stimulate intra-bone tumor invasion. These findings suggest that intercellular communication between osteoblasts and cancer cells plays a pivotal role in its disease progression [3,4].

Ameloblastoma is a rare neoplasm that represents about 11–18% of all jaw tumors and cysts. World Health Organization defines ameloblastoma as an odontogenic tumor in the bone jaw with multiple histologic variants [[5], [6], [7], [8], [9], [10]]. Ameloblastoma shows specific characteristics of extensive bone resorption in its clinical appearance. Ameloblastoma is also associated with a high recurrence rate if treated improperly [5,[9], [10], [11], [12], [13]]. Attempts have recently been made to clarify the underlying pathogenesis of ameloblastoma. Current studies on ameloblastoma focus on the bone resorption process (osteoclastogenesis) and the roles of other cells in the bone environment such as stromal cells. We found that ameloblastoma cells and stromal fibroblasts behave interactively via the regulation of inflammatory cytokines to create a microenvironment that leads to the extension of ameloblastoma [14]. However, until recently, there has been limited research on interactions between ameloblastoma cells and osteoblasts [15]. This study aimed to clarify interactions between ameloblastoma cells and osteoblasts that are associated with the pathogenesis of ameloblastoma.

## 2 Materials and methods

### 2.1 Cell culture

AM-3 ameloblastoma cells were maintained with a defined keratinocyte serum-free medium (D-KSFM, Thermo Fisher Scientific, Tokyo, Japan), as previously described [16]. MC3T3-E1 Subclone 4 cells, a murine pre-osteoblast cell line, were cultured in Minimum Essential Medium-Alpha Modification (α-MEM) without ascorbic acid and supplemented with 10% fetal bovine serum (FBS).

### 2.2 Preparation of MC3T3-E1-derived conditioned medium (CM)

A total of 1 × 10^5^ MC3T3-E1 cells were cultured in a 100 mm-diameter dish with α-MEM supplemented with 10% FBS. After 24 h, the medium was replaced with a differentiation medium (growth medium plus 50 μg/mL ascorbic acid and 10 mM β-glycerophosphate). MC3T3-E1 cells were grown to mature stage differentiation for 14 days. The differentiation medium was exchanged every three days, and on the 14th day, the CM was collected and stored at −80 °C until further use.

### 2.3 Preparation of AM-3-derived CM

A total of 3 × 10^5^ AM-3 cells were cultured in a 60 mm-diameter dish with 3 mL of D-KSFM. After 24 h, CM was collected and stored at −80 °C for cytokine production assay experiments.

### 2.4 Indirect cocultures of AM-3 and MC3T3-E1 cells

Semi-confluent MC3T3-E1 cells were cultured in a 35 mm-diameter dish for 24 or 72 h. The cells were treated with D-KSFM (50% (v/v)), AM-3 CM (50% (v/v)), or AM-3 CM (50% (v/v)) supplemented with recombinant IL-1Ra (100 ng/mL) (FUJIFILM Wako Chemicals, Japan), which is an IL-1 receptor antagonist. The culture media were collected and stored at −80 °C for cytokine assay using ELISA.

Semi-confluent AM-3 cells were cultured in a 35 mm-diameter dish for 24 or 72 h. The cells were treated with α-MEM supplemented with 10% FBS (50% (v/v)), or MC3T3-E1 CM (50% (v/v)). The culture media were collected and stored at −80 °C for MMP assay using ELISA.

### 2.5 DNA microarray

DNA microarray analysis using RNA were done as previously described [17]. The gene expression profiling was analyzed by Simple Array Analyzer (developed by iAnalyze (Kagoshima, Japan)) according to the user manuals.

### 2.6 Real-time RT-PCR

Total RNA was extracted from cells and reverse-transcribed as described previously [17]. Gene expression of cytokine mRNA was estimated by real-time RT-PCR using LightCycler TaqMan Master, LightCycler 1.5, and LightCycler software version 3.5 (Roche Diagnostics, Tokyo, Japan) according to the manufacturer's instructions. Expression of each mRNA was normalized using β-actin as a loading control. All primers used in this study were described as previously [17].

### 2.7 ELISA

The concentrations of IL-6, monocyte chemoattractant protein-1 (MCP-1), regulated on activation, normal T-cell expressed and secreted (RANTES), and matrix metalloproteinases (MMPs) were analyzed by the chemiluminescence-based Q-Plex™ Mouse Cytokine Array and Human MMP (Quansys Biosciences, Logan, UT, USA) using the manufacturer's protocol. Digital images were acquired and analyzed as described previously [17].

### 2.8 Western blot analysis

CM were centrifuged and aliquots were separated using 8% sodium dodecyl sulfate-polyacrylamide gel electrophoresis, and analyzed using anti-hMMP-2 antibody (F-68, Kyowa Pharma Chemical Co., Ltd., Toyama, Japan). Accumulation of MMP-2 proteins were analyzed as described previously [16].

### 2.9 Proliferation assay

AM-3 cells (1 × 10^5^ cells) were seeded in a 60 mm-diameter dish with D-KSFM in the presence or absence of MC3T3-E1 CM (50% (v/v)). Cells were counted on days 1, 3, and 5.

### 2.10 Migration assay

A modified Boyden-chamber (tissue culture treated, 6.5 mm in diameter, 10 μm thickness, 8 μm pores; Transwell® (Corning, Inc., Corning, NY, USA)) was used as previously described [17]. Cells (0.5 × 10^4^ cells) were suspended in D-KSFM and applied to the upper chamber. On the lower chamber, α-MEM + FBS medium were applied as a control group or MC3T3-E1 CM as a treatment group. After 1 or 2 h, the following protocol were done as previously described [17].

### 2.11 Statistical analyses

Statistical analyses were carried out using the parametric independent *t*-test with **Microsoft® Excel.**

## 3 Results

### 3.1 Interactions between ameloblastoma cell and osteoblasts triggered inflammatory cytokine production by osteoblasts

**Fig. 1:**
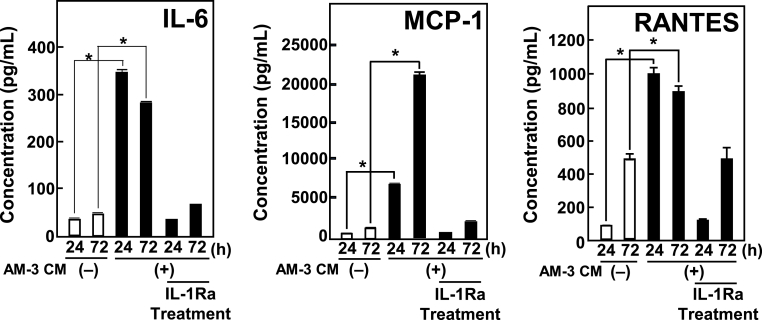
Productions of IL-6, MCP-1, and RANTES by MC3T3-E1 cells were stimulated by AM-3 CM. After MC3T3-E1 cells were treated with D-KSFM (as a control (−)), AM-3 CM, and AM-3 CM in the presence of IL-1Ra (100 ng/mL) for 24 or 72 h, concentrations of IL-6, MCP-1, and RANTES protein in CM were quantified by the Q-Plex™ ELISA kit. Results are means ± SE from three independent experiments. Asterisks (*) indicate statistically significant results, *p* < 0.05.

The presence of AM-3 CM induced IL-6, MCP-1, and RANTES secreted from MC3T3-E1 cells compared with unstimulated MC3T3-E1 cells (Fig. 1).

Previously we established a follicular ameloblastoma cell line (AM-3) and AM-3 highly expressed IL-1α [16,17]. We confirmed that similar high expressions of IL-1 genes by real-time PCR and ELISA (Supplementary Fig. 1 and Supplementary Table 1) in the condition of this study.

Furthermore, we examined the effects of an IL-1 receptor antagonist (IL-1Ra) on inflammatory cytokine production by MC3T3-E1 osteoblast cells. Administration of the IL-1Ra suppressed the production of IL-6, MCP-1, and RANTES by MC3T3-E1 osteoblasts stimulated with AM-3 CM (Fig. 1).

### 3.2 Interactions between ameloblastoma cells and osteoblasts triggered MMP-2 production by ameloblastoma cells

**Fig. 2:**
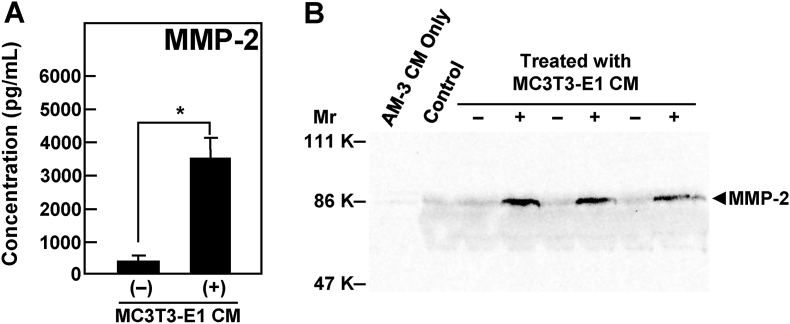
Secretion of MMP-2 from AM-3 cells stimulated by MC3T3-E1 CM. (A) The AM-3 cell-derived production of MMP-2 protein in CM was quantified by the Q-Plex™ ELISA kit. Results are means ± SE from three independent experiments. An asterisk (*) indicates statistically significant results, *p* < 0.05. (B) The protein expression of MMP-2 in CM of AM-3 ameloblastoma cells treated without (−) or with (+) MC3T3-E1 CM is shown by Western blot. AM-3 only, CM of AM-3 cells cultured with D-KSFM for 72 h. Control, a mixture of D-KSFM (50% (v/v)) and MC3T3-E1 CM (50% (v/v)) incubated at 37 °C for 72 h.

ELISA and Western blot analysis were used to examine the effect of MC3T3-E1-derived CM on the expressions of MMPs by ameloblastoma cells. MMP-2 expression by AM-3 cells treated with MC3T3-E1 CM was upregulated compared with unstimulated AM-3 cell as shown in Fig. 2. However, the expressions of MMP-1, MMP-3, and MMP-9 were not modulated by MC3T3-E1 CM. The expressions of MMP-7 and MMP-13 were not detected (Supplementary Table 2). In addition, microarray study showed that MMP-14 (MT1-MMP) showed moderately increased expression (2.297-fold) in the AM-3 ameloblastoma cells treated with MC3T3-E1 CM compared to the AM-3 ameloblastoma cells alone.

### 3.3 Osteoblast CM stimulated the proliferation of ameloblastoma cells

**Fig. 3:**
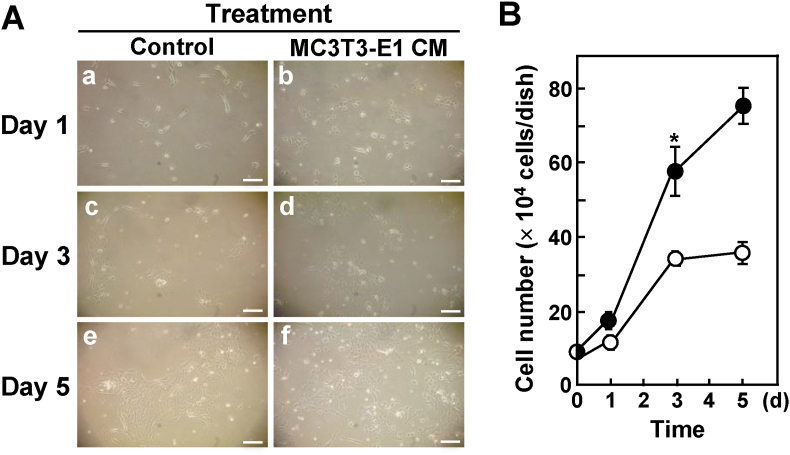
The proliferations of AM-3 cells were stimulated by MC3T3-E1 CM. (A) Representative microscopic images. AM-3 cells were seeded and cultured in the presence of α-MEM (50% (v/v)) as a control (a, c, e) or in the presence of MC3T3-E1 CM (50% (v/v)) (b, d, f). (a and b), 1-day cultured. (c and d), 3-day cultured. (e and f), 5-day cultured. Scale bar, 100 μm. (B) Cell number growth data. Results are means ± SE from three independent experiments. An asterisk (*) indicates statistically significant results, *p* < 0.05. Closed circles (●) show AM-3 cells treated with MC3T3-E1 CM. Open circles (○) show AM-3 cells treated with α-MEM/FBS (as a control).

AM-3 cells grew faster on the third day of culture in the presence of MC3T3-E1 CM compared with AM-3 cells treated with α-MEM as a control (Fig. 3). We previously reported that IL-6 itself accelerated the growth of AM-3 cells [17]. However, IL-6 neutralizing antibody showed no effects on the proliferation of AM-3 cells stimulated by MC3T3-E1 CM (data not shown).

### 3.4 Osteoblast CM acted as a chemoattractant and regulated cellular motility of ameloblastoma cells

**Fig. 4:**
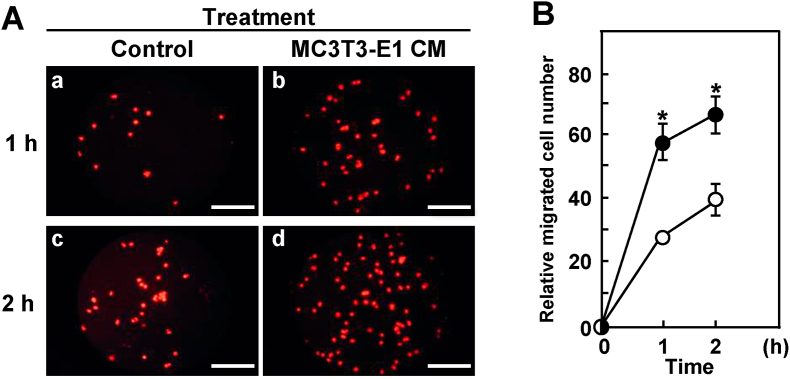
Cellular motility of AM-3 cells is accelerated by MC3T3-E1 CM. (A) Representative fluorescent images. AM-3 cells migrated for 1 h (a and b) or 2 h (c and d). (a and c), AM-3 cells were seeded and cultured in the presence of α-MEM (50% (v/v)) as a control. (b and d), AM-3 cells were seeded and cultured in the presence of MC3T3-E1 CM (50% (v/v)). Scale bar, 100 μm. (B) Relative migrated cell number data. Results are means ± SE from three independent experiments. Asterisks (*) indicate statistically significant results, *p* < 0.05. Closed circles (●) show AM-3 cells treated with MC3T3-E1 CM. Open circles (○) show AM-3 cells treated with α-MEM/FBS (as a control).

We examined whether MC3T3-E1 CM affects the cellular motility of ameloblastoma cells. The cellular motility of AM-3 cells was significantly increased by the treatment with MC3T3-E1 CM (Fig. 4).

## 4 Discussion

In terms of the tumor and bone microenvironment, bone is a fertile environment for tumor growth because it has many sources of growth factors, neovascularization factors, cytokines, chemokines, and other soluble proteins [18]. These factors facilitate tumor cell colonization, growth, or survival through evading the immune surveillance system. Furthermore, these factors also contribute to destruction of the extracellular matrix (ECM) and the creation of nutrient access for tumor cells.

It was reported that osteoblast-derived factors act through paracrine or autocrine manners between osteoblasts and cancer cells. The amount of osteoblast-derived cytokines increased in the presence of breast cancer cells-derived CM or direct contact with a metastatic breast cancer cell variant [3,19].

Several studies reported that some cytokines were secreted from ameloblastoma cells, including IL-1α, IL-1β, IL-6, and TNF-α [20,21]. This study found that MC3T3-E1 osteoblast cells produced IL-6, MCP-1, and RANTES in the presence of ameloblastoma CM. Furthermore, we showed that ameloblastoma cells stimulated osteoblasts to produce large amounts of cytokines in an IL-1-dependent manner.

These cytokines are known to be involved in the regulation of bone remodeling under physiological and pathological conditions. IL-6 is a cytokine with osteolytic activity and has been implicated in the growth and expansion of ameloblastoma by promoting tumor growth and modifying bone remodeling. IL-6 regulates the differentiation of osteoclast progenitor cells into mature osteoclasts and also directly stimulates RANKL production in bone [[20], [21], [22]]. Furthermore, the cytokine production induced due to sustained pathologic conditions can elicit increased osteoclastogenesis [3].

Chemokines, including MCP-1 and RANTES, also have a role in regulating bone remodeling under physiological or pathological conditions. Chemokines play divergent roles in various phases of pathogenesis and immune reactions. They control the migration, localization, and functions of immune cells as chemoattractants during inflammation [23,24]. MCP-1 is produced by osteoblasts in response to PTH and inflammatory cytokines. It also plays a crucial role in bone remodeling through its involvement in osteoclast differentiation and maturation. MCP-1 is a ligand for CCR2, which has a major effect on osteoclasts. RANTES binds to CCR1 and CCR2, which activate osteoclastogenesis. Chemokines and their receptors work not only under these physiological conditions but also in cancer-microenvironments to stimulate osteoclastogenesis and invasion [[25], [26], [27], [28]]. These findings suggest that ameloblastoma cells and osteoblasts affect each other in the bone microenvironment to promote the bone remodeling process.

The invasion of surrounding healthy tissues by tumor cells is one of the essential steps in tumor progression. This complex phenomenon requires simultaneous processes such as decreased adhesion, increased motility, proteolysis, and resistance to apoptosis [29]. Proteolytic enzymes play roles in degrading the surrounding ECM. Matrix metalloproteinases (MMPs) are zinc metalloenzymes involved in ECM remodeling. A previous report postulated that in physiologic condition, MMPs, at least MMP-2, MMP-3, MMP-9, MMP-13 and MMP-14 are important for remodeling process [30]. MMP-2 and MMP-9 were identified as target genes of Wnt signaling and play a role in the degradation of the ECM of the bone [31]. Several studies also demonstrated that MMPs like MMP-1, MMP-2, MMP-7, MMP-9 and MMP-14 are involved in ECM degradation in the invasion of ameloblastoma [16,21,22,[32], [33], [34]]. While AM-3 cells spontaneously produced high amount of MMP-9 and had potential to increase MMP-9 production in response to Wnt-3a [16], this study demonstrated that AM-3 ameloblastoma cells showed higher expression of MMP-2 by the treatment of MC3T3-E1 osteoblast CM. In addition, our microarray study suggested that MMP-14 showed moderately increased expression in the AM-3 ameloblastoma cells stimulated by MC3T3-E1 CM compared with the AM-3 ameloblastoma cells alone.

These findings indicated that ameloblastoma cells could receive signals for production of MMPs from tumor-associated bone tissues, which may contribute to the degradation of the bone matrix. MMP-2 and MMP-9 belong to gelatinases and digest the denatured collagens and gelatins. MMP-9 also contributes to wound healing suppression by the continuous breakdown of collagen [35]. Both of the basal expressions of MMPs family and induced expressions of MMPs by intercellular communication in bone tumor microenvironment will lead to the degradation of ECM. The degradation of ECM by the MMP family removes the physical barriers which protect tumor invasion. It also releases numerous biologically active molecules, such as TGF-β in the bone substrates [[35], [36], [37]].

Previous studies showed that osteoblasts released proteins that increase the secretion of soluble factors to promote tumor growth, migration, and invasion capability of cancer cells [3,19,38,39]. In malignant tumors, MCP-1 and RANTES were reported to promote tumor cell proliferation, migration, and metastasis [[40], [41], [42]]. Findings on this study showed that MC3T3-E1 osteoblast cells secreted IL-6, MCP-1, and RANTES in response to the intercellular communication between ameloblastoma cells and osteoblasts. Besides that, the MC3T3-E1 CM of the unstimulated condition itself showed a stimulatory effect on proliferation and migration capability of AM-3 ameloblastoma cells, whereas IL-6, MCP-1 and RANTES were not secreted in the unstimulated MC3T3-E1 CM. These findings imply that MC3T3-E1 cells produced other soluble factors that promote the proliferation and migration capability of AM-3 ameloblastoma cells rather than IL-6, MCP-1, or RANTES. Taken together, these findings demonstrated that MC3T3-E1 osteoblasts not only have a basal activity on proliferation and migration of AM-3 ameloblastoma cells but also produce several induced-cytokines in response to the intercellular communication between osteoblasts and ameloblastoma cells. Previous reports postulated that IL-6, MCP-1, RANTES, and MMPs might involve in activating osteoclastogenesis, which in turn will support the tumor progression [3,23,24,26]. The same mechanism may be assumed that these cytokines and MMPs may work in concert and modify the bone tumor microenvironment that will contribute to ameloblastoma tumor cell growth, migration, and bone invasion.

Wilms’ tumor 1 (WT1) have been primarily considered in the development of Wilms’ tumor. WT1 also reported it plays multiple roles in development, tissues homeostasis, and disease [43,44]. A previous study postulated that WT1 was involved in the regulation of osteoclastogenesis in an osteoclast precursor cell line [45]. Furthermore, in conditions such as asthma and chronic obstructive pulmonary disease, WT1 was reported to act as a repressor of MMP-9 [46]. In contrast, in most solid tumors, WT1 acted as an oncogenic factor that promote tumorigenesis [44,47,48]. Moreover, the expressions of WT1 has also been reported as a significant oncogene to the pathogenesis in various histological type of ameloblastoma [49,50]. However, the microarray data in this study showed no differences (fold change 1.023) in the expressions of WT1 between monoculture of AM-3 ameloblastoma cell and AM-3 cell treated with MC3T3-E1 CM.

Recently, the IL-1 receptor antagonist has been widely used to treat patients with several diseases, e.g., rheumatoid arthritis, diabetes type 2, and osteoarthritis [51]. We previously reported that the productions of IL-6 and IL-8 were induced in stromal fibroblasts stimulated with AM-3 ameloblastoma-derived IL-1α [17]. This study also showed that ameloblastoma cells stimulated osteoblasts to produce cytokines in an IL-1-dependent manner. These findings suggest that IL-1 is a key mediator of ameloblastoma to control its microenvironment and local invasion. Ameloblastoma and surrounding cells, including fibroblasts and osteoblasts, interacted with each other through cytokines and metalloproteinases to create a pathological microenvironment similar in the case of malignant tumors (Supplementary Fig. 2).

In conclusion, ameloblastoma cells stimulated osteoblasts to produce IL-6, MCP-1, and RANTES, which can promote tumor growth and modify the bone remodeling process by inducing osteoclastogenesis. Osteoblast-derived factors induced the production of MMP-2 by ameloblastoma cells which degrade ECM. Furthermore, unidentified factors from osteoblast CM accelerated the proliferation and migration of AM-3 ameloblastoma cells.

Proper understanding of the pathogenesis involved in ameloblastoma will help develop new therapeutic approaches. Neutralizing agents for IL-1 or MMP-2 may have therapeutic use to control ameloblastoma locally.

## Authors contribution

EC, TF and SK contributed to the concept underlying the study and designed the study. EC,YO,HK and MK acquired the data. EC and HK contributed to the analysis and interpretation of the data. EC drafted the manuscript. EC, TF, HK, MI, TK, NN, and SK revised the content of the manuscript. All authors read and approved the final manuscript.

## Declaration of competing interest

The authors declare that they have no known competing financial interests or personal relationships that could have appeared to influence the work reported in this paper.

## Abbreviations

α-MEM: Minimum essential medium-alpha modification
CM: Conditioned medium
D-KSFM: Defined keratinocyte serum-free medium
FBS: Fetal bovine serum
IL: Interleukin
IL-1Ra: IL-1 receptor antagonist
MCP-1: Monocyte chemoattractant protein-1
MMPs: Matrix metalloproteinases
RANTES: Regulated on activation, normal T-cell expressed and secreted
WT1: Wilms’ tumor 1
