# Supplementary figures and images for: Intercellular signaling between ameloblastoma and osteoblasts
Source: Biochem Biophys Rep. 2022 Feb 18;30:101233. doi: 10.1016/j.bbrep.2022.101233 (PMC8861578; doi:10.1016/j.bbrep.2022.101233)

## Slide 1
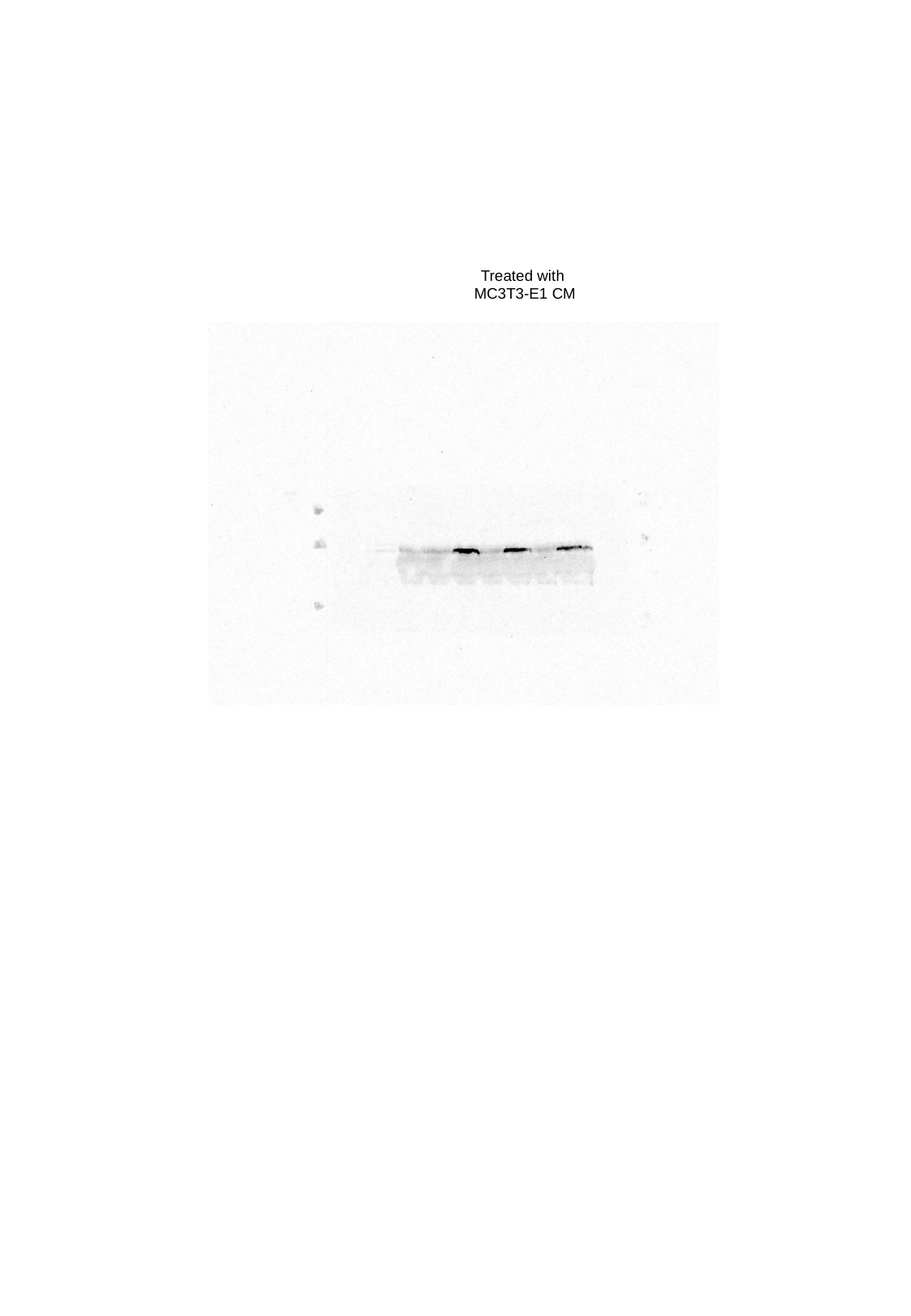

Treated with
MC3T3-E1 CM

Supplement: Multimedia component 1 [file mmc1.pptx]
