# Supplementary material for: Intercellular signaling between ameloblastoma and osteoblasts
Source: Biochem Biophys Rep. 2022 Feb 18;30:101233. doi: 10.1016/j.bbrep.2022.101233 (PMC8861578; doi:10.1016/j.bbrep.2022.101233)
Supplement: Multimedia Component 2 [file mmc2.pptx]

## Slide 1
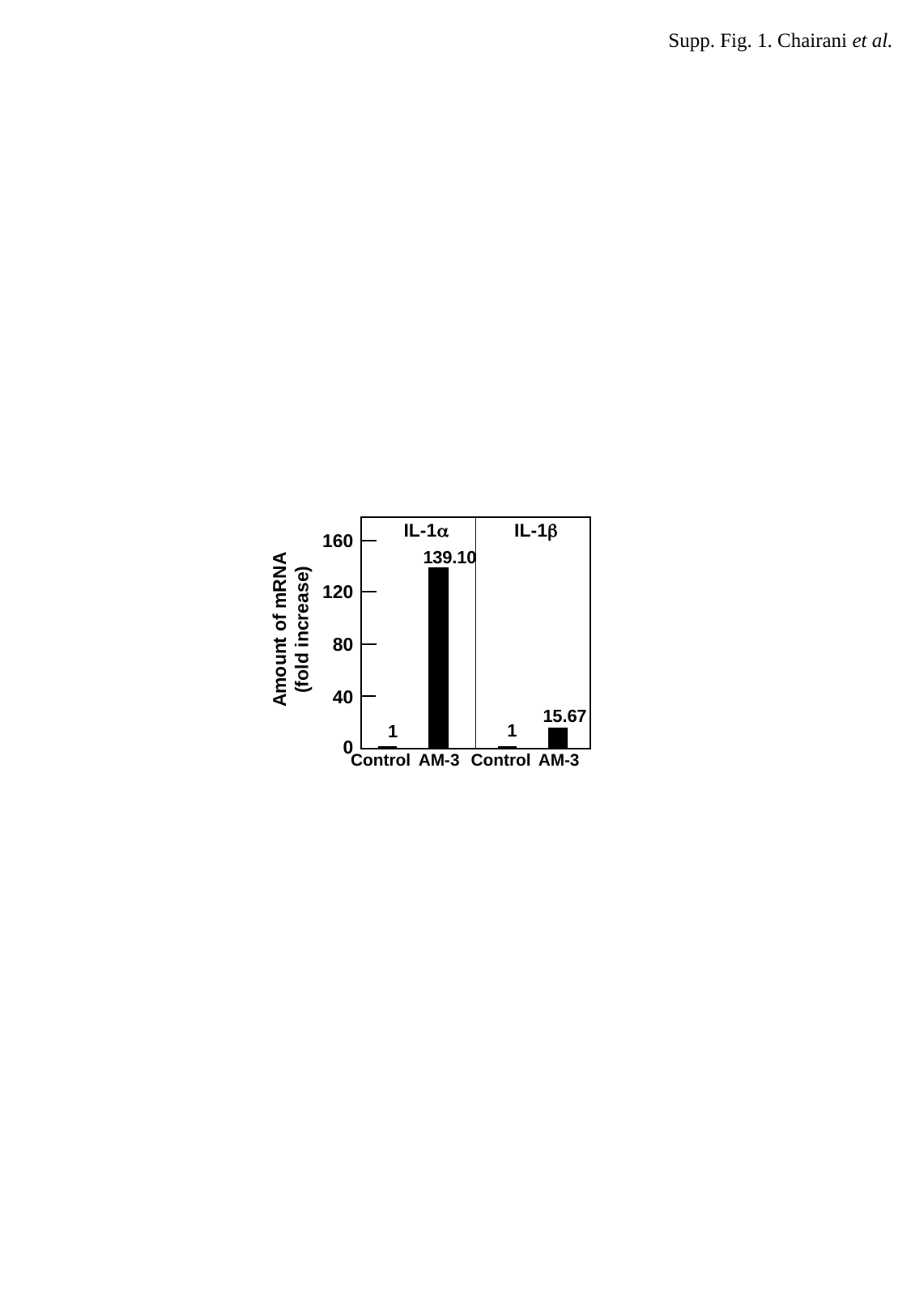

Supp. Fig. 1. Chairani et al.
IL-1a
IL-1b
139.10
15.67
1
1
160
Amount of mRNA
(fold increase)
120
80
40
0
Control
AM-3
Control
AM-3

## Slide 2
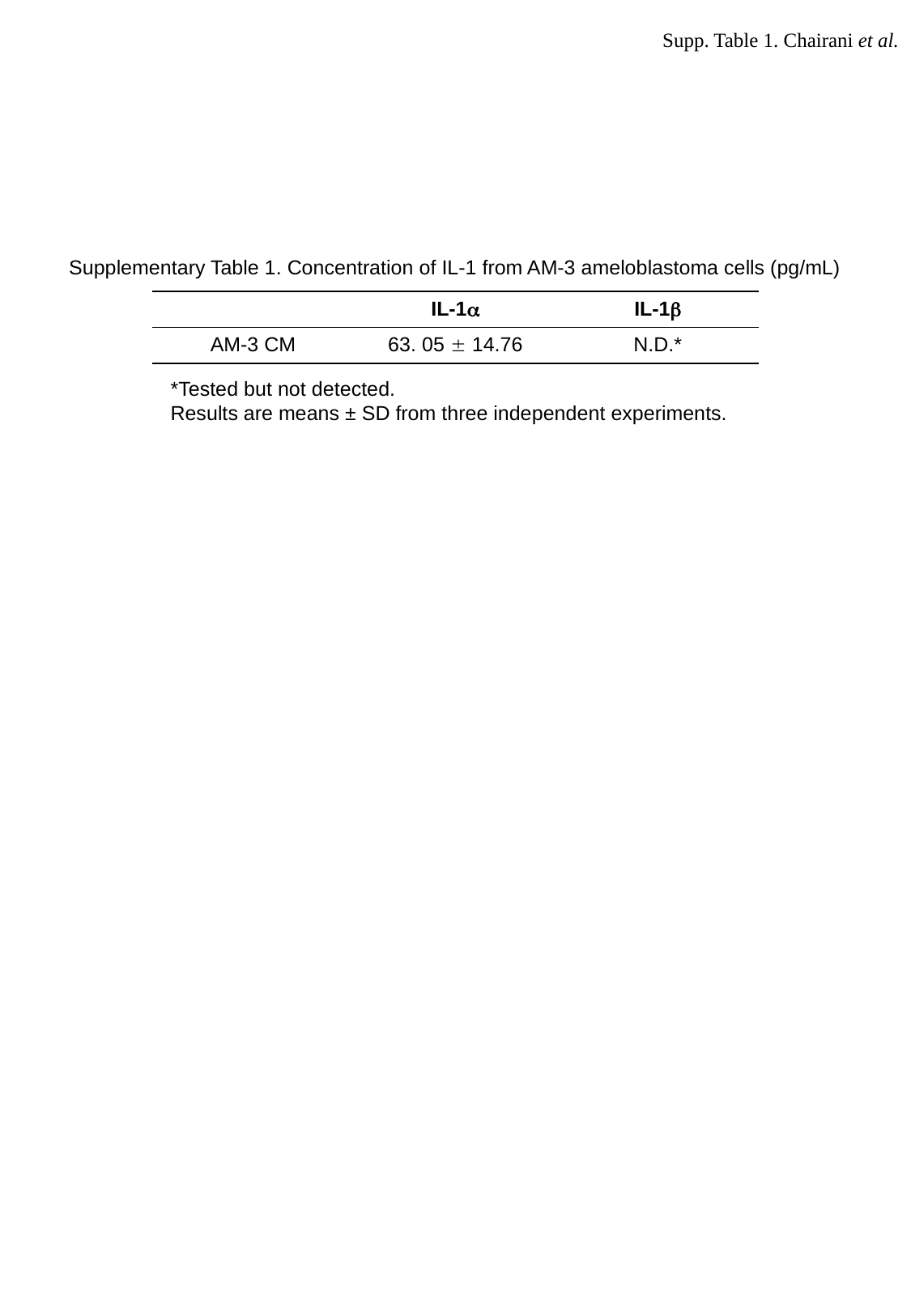

Supp. Table 1. Chairani et al.
Supplementary Table 1. Concentration of IL-1 from AM-3 ameloblastoma cells (pg/mL)
| | IL-1a | IL-1b |
| --- | --- | --- |
| AM-3 CM | 63. 05 ± 14.76 | N.D.\* |
*Tested but not detected.
Results are means ± SD from three independent experiments.

## Slide 3
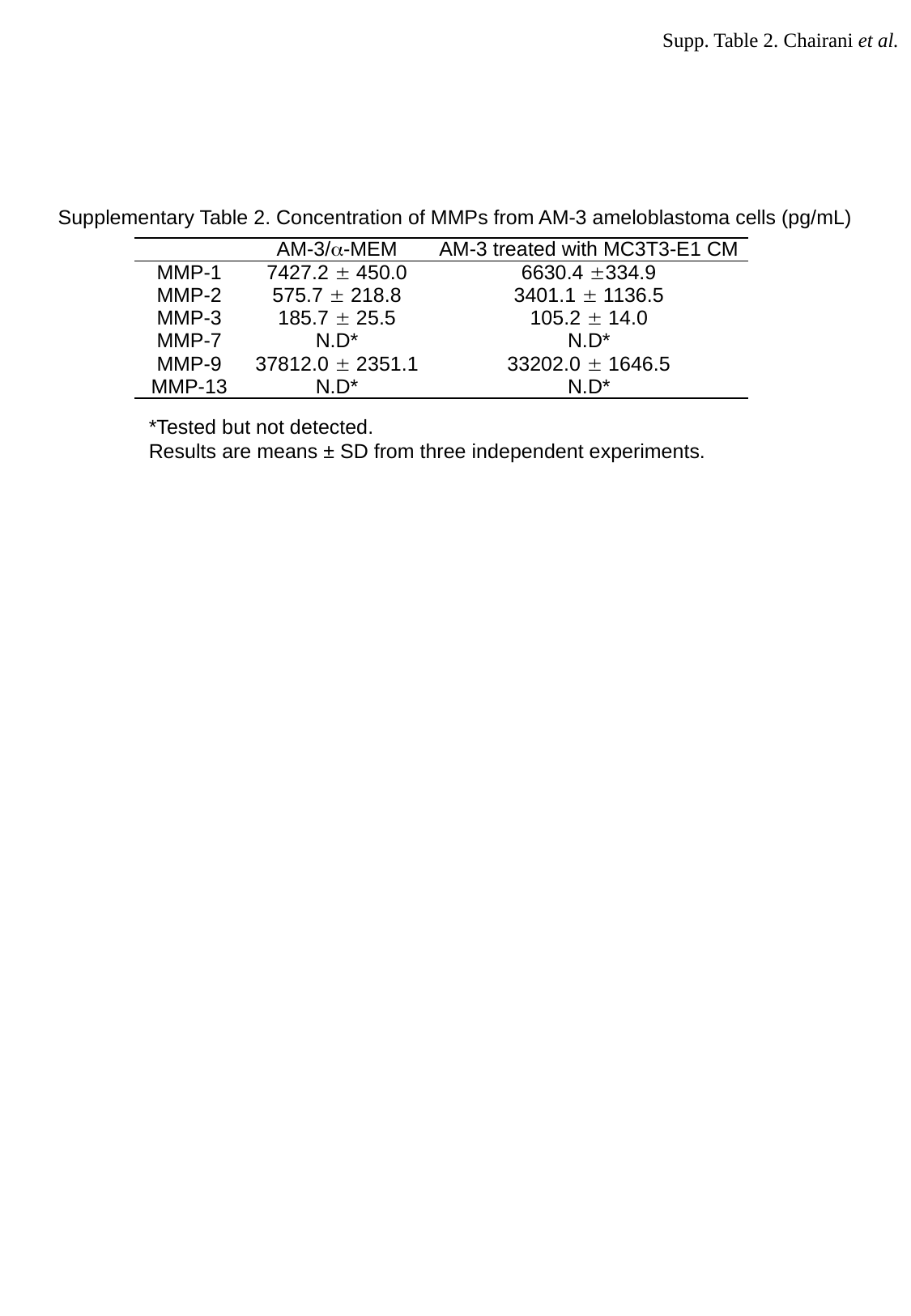

Supp. Table 2. Chairani et al.
Supplementary Table 2. Concentration of MMPs from AM-3 ameloblastoma cells (pg/mL)
| | AM-3/a-MEM | AM-3 treated with MC3T3-E1 CM |
| --- | --- | --- |
| MMP-1 | 7427.2 ± 450.0 | 6630.4 ±334.9 |
| MMP-2 | 575.7 ± 218.8 | 3401.1 ± 1136.5 |
| MMP-3 | 185.7 ± 25.5 | 105.2 ± 14.0 |
| MMP-7 | N.D\* | N.D\* |
| MMP-9 | 37812.0 ± 2351.1 | 33202.0 ± 1646.5 |
| MMP-13 | N.D\* | N.D\* |
*Tested but not detected.
Results are means ± SD from three independent experiments.

## Slide 4
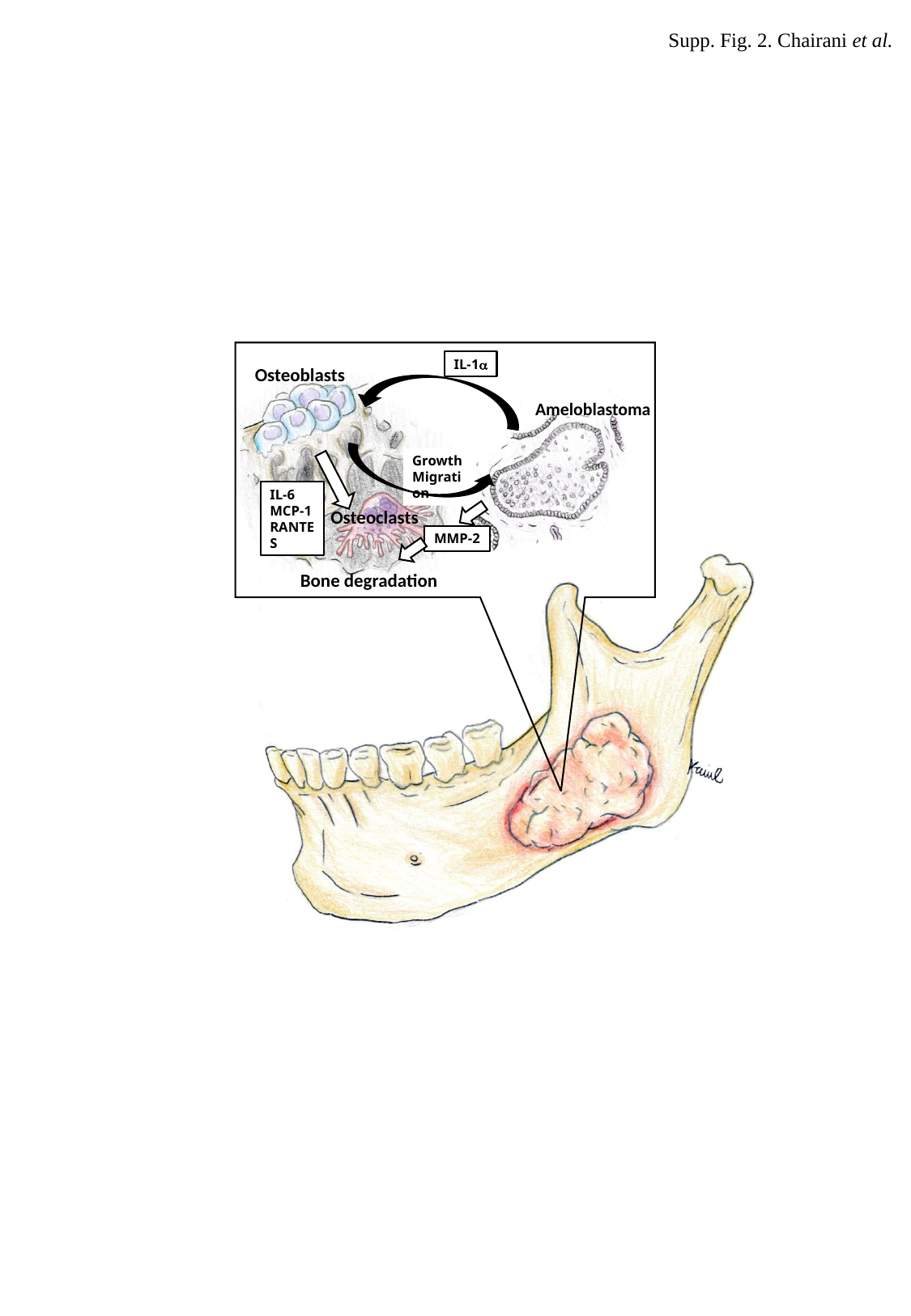

Supp. Fig. 2. Chairani et al.
IL-1a
Ameloblastoma
Growth
Migration
IL-6
MCP-1
RANTES
Osteoblasts
Osteoclasts
MMP-2
Bone degradation
